# Ending the (Cell) wall metaphor in microbiology

**DOI:** 10.1016/j.tcsw.2022.100087

**Published:** 2022-11-10

**Authors:** Arturo Casadevall, Neil A.R. Gow

**Affiliations:** a School of Medicine, Department of Molecular Microbiology and Immunology, Johns Hopkins, Bloomberg School of Public Health, 615 N. Wolfe Street, Baltimore, MD 21205, USA; b Medical Research Council Centre for Medical Mycology, University of Exeter, Geoffrey Pope Building, Stocker Road, Exeter EX4 4QD, UK

**Keywords:** Bacteria, Cell wall, Surfaces, Fungi, Organelle

## Abstract

• The outer surfaces of fungi and bacteria are highly dynamic, flexible, regulated and adaptable structures.
• The term “cell wall” is conceptually limiting and insufficient to adequately describe the outer surface of bacteria and fungi.
• The Greek term plégma or in Latin word “reticulum” better capture the nature of the cell wall.

Words matter for they are essential to the symbolic communication that is unique to our species. The way we use words shapes our thoughts and actions. However, words can also be limiting when used to refer to complex topics in topics. For example, one of the authors has argued that the word ‘pathogen’ is limiting our understanding of microbial pathogenesis and may even be hindering progress in the field (Casadevall and Pirofski, 2014). Similarly, the intracellular pathogen concept probably hindered progress in immunology since the word “intracellular” focused thinking into the duality of microbes being intracellular or extracellular despite a nuanced complexity in microbial lifestyles whereby most had phases in and out of host cells (Casadevall and Fang, 2020). Here we consider the limitations of the term ‘cell wall’ in microbiology and suggest the wall metaphor could be having a similar detrimental effect by creating a false representation in our minds that limits understanding and skews the direction of experimental work.

The concept of a wall to contain cellular contents in a rigid structure dates the 17th century when Robert Hook named the structures delimiting plant cells as walls because they reminded him of the rows of small cells in a monastery. Before going further, it is worthwhile considering the definition of the word “wall”. Looking in dictionaries it is quickly apparent that the word wall has many meanings that depend on context. The encyclopedia Britannica defines wall as ‘structural element used to divide or enclose, and, in building construction, to form the periphery of a room or a building’ (https://www.britannica.com/technology/wall), which partially fits its role in microbiology given that it divides and encloses fungal, plant and certain bacterial cells. However, walls also connote the defensive structures as Hadrian’s Wall and the Great Wall of China with the primary purpose of keeping invaders out, a meaning less applicable in microbiology where Gram positive walls are insufficient to prevent bacteriophage infection and fungal walls are permissive to such large structures as liposomes (Walker et al., 2018) and even bacterial invaders (Moebius et al., 2014).

But the analogy to a wall also fails when one considers the compositions of structural and cell walls. Structural walls are rigid and generally composed of a limited number of components, which may be metal, masonry or wood while microbial cell walls are made of a remarkably complex assembly of polysaccharides, proteins, lipids and pigments such as melanin. Structural walls are static and generally immovable while microbial walls are living structures that can change rapidly as evident by the rearrangement that can follow such events as the yeast to hyphal transition in fungi. In fact, the fungal cell wall manifest considerable elastic properties that is regulated throughout the cell cycle and which changes depending on the growth conditions, and elasticity is modulated by remodeling enzymes (Ene et al., 2015, Zhao et al., 2005). Indeed, the cell wall is one of the most regulated structures in the microbiological world, and in fungi it has been estimated that perhaps a fifth of the genome contributes to its assembly and maintenance (Verstrepen et al., 2004). Fungal cell walls are therefore not one structure, but many structures depending on the shape of the cell, the nature of the external environment and the response to various imposed physical and biochemical stresses and strains (Gow and Lenardon, 2022).

The wall metaphor for the outer cellular structural in fungi and Gram-positive bacteria connotes the impression of rigidity and impenetrableness. We can think of at least one example where this view hindered progress in the field and that is in the appreciation that these organisms made extracellular vesicles. Whereas such structures were known in Gram negative bacteria where they are assumed to arise from the outer cellular membrane the presence of a cell wall in fungi and Gram-positive bacteria was believed to prevent the release of such structures since these were presumably too large to pass through cell wall pores. Hence, there was no systematic search for these structures in Gram positive bacteria or fungi and when vesicle like structures were seen in electron micrographs these were usually dismissed as lipid artifacts since release pass the cell wall was considered ‘impossible’ (Bose et al., 2020). So, when it was reported that fungi made extracellular vesicles in 2007 (Rodrigues et al., 2007) and later in Gram positive bacteria (Rivera et al., 2020, Lee et al., 2009) and mycobacteria (Prados-Rosales et al., 2011) the initial reception was muted simply because people had trouble believing that such structures could cross the fungal and bacterial cell walls. One could argue that had microbiologists been on a non-wall frame of mind that these discoveries could have happened earlier. Today it is accepted that cell-walled microorganisms produce extracellular vesicles and this field has exploded as these have been implicated in biofilm formation, delivery of virulence factors and microbial communication.

AmBisome is a commercial antifungal agent that incorporates the insoluble polyene amphotericin B into liposomes that can deliver the antifungal payload to the cell membrane where it causes irreversible damage. Our research groups showed that these liposomal vesicles do not have to disassemble to transit through the wall despite having a size ten times that of its estimated chemical porosity (Walker et al., 2018). Indeed, these liposomes, which are similar in size to secretory extracellular vesicles, could even transport encapsulated colloidal gold particles through the cell wall. Therefore, vesicular traffic through the cell wall occurs in both outward and inward directions, challenging the appropriateness of the term “wall” as an adequate metaphor for the cell surface layer.

Moving away from the wall concept could make it easier for scientists to focus on the remarkable aspects of these structures, including their ability to be rapidly remodeled and its permeability to large structures such as bacteria and vesicles. When one considers the intricate meshwork of polysaccharide and other components that comprise this outer structure its ability to be rapidly reshaped and precisely reshaped for such critical cell processes as budding, hyphal and other morphological transitions and vesicular permeability provides questions that are currently unanswered providing major problems for future study. In fact, the view of cell wall as a rigid structure may have more to do with the lack of good techniques for addressing these questions. In this regard, the alkaline extraction protocol that has been used for decades to isolate cells leaves behind a structural corpse of chitin and alkali insoluble glucans that has little or no relevance to the living structure. Although the resulting particles, such as zymosan, have proven invaluable in immunological discovery of recognition receptors their apparent rigidity does not reflect the flexibility of living cell structures and we can imagine how these could exacerbate mistaken impressions on the nature of the cell wall that contribute to erroneous wall concept.

Abandoning the wall name and metaphor for the outer structures that surround fungi, Gram positive bacteria and mycobacteria raise the question of how the cell wall should be renamed. We considered the word ‘organelle’ but this does not work since all other organelles are membraned bound structures, which does not apply to structure currently known as the cell wall. The cell wall is much more of living mesh than a wall. One option is to rely on the Greek language, which has supplied so many key words to language of science. Mesh in Greek is *plégma* and the cell wall could be renamed the cell plégma. In Latin mesh is “reticulum”, which works except that this term already has various uses in anatomy. Arthropods such as insects and crustaceans have an “exoskeleton” which could be an alternative term. The arthropod exoskeleton could be argued as being a less regulated or complex structure than fungal or bacterial walls. Like these complex animals some fungi are even able molt whole intact cell wall layers (Lopes-Bererra et al., 2018) and microbial surfaces have many other functions than to determine shape. None of these alternative terms fully captures the subtleties and multifunctional natures of microbial cell surfaces.

## Conclusion

In summary, we suggest a need to end the cell wall metaphor in microbiology because it does not accurately reflect the composition of the structure or its true role in partitioning and orchestrating the living cell. Its continued use may inhibit research progress by subtly affecting how we think about its purpose, composition and function. Pragmatically, the term wall may be retained in our literatures, but that should not obscure the remarkable intricacy, flexibility and adaptability of this series of structures, or undermine efforts to understand how they work.

## Declaration of Competing Interest

The authors declare the following financial interests/personal relationships which may be considered as potential competing interests: On behalf of the authors: Arturo Casadevall and Neil A.R. Gow, Given his role of Editor-in-Chief of the journal, Neil Gow had no involvement in the peer review of this article and has had no access to information regarding it’s peer review. Full responsibility for the editorial process for this article was delegated to Gurdyal Besra.

## References

[b0005] Bose S., Aggarwal S., Singh D.V., Acharya N. (2020). Extracellular vesicles: An emerging platform in gram-positive bacteria. Microbial Cell..

[b0010] Casadevall A., Fang F.C. (2020). The intracellular pathogen concept. Mol. Microbiol..

[b0015] Casadevall A., Pirofski L.A. (2014). Microbiology: Ditch the term pathogen. Nature.

[b0020] Ene I.V., Walker L.A., Schiavone M., Lee K.K., Martin-Yken H., Dague E. (2015). Cell wall remodeling enzymes modulate fungal cell wall elasticity and osmotic stress resistance. MBio..

[b0025] Gow N.A.R., Lenardon M.D. (2022). Architecture the dynamic fungal cell wall. Nat. Rev. Microbiol..

[b0030] Lee E.Y., Choi D.Y., Kim D.K., Kim J.W., Park J.O., Kim S., Kim S.H., Desiderio D.M., Kim Y.K., Kim K.P. (2009). Gram-positive bacteria produce membrane vesicles: proteomics-based characterization of Staphylococcus aureus-derived membrane vesicles. Proteomics.

[b0035] Lopes-Bezerra L.M., Walker L.A., Niño-Vega G., Mora-Montes H.M., Neves G.W.P., Villalobos-Duno H., Barreto L., Garcia K., Franco B., Martínez-Álvarez F.A., Munro C.A., Gow N.A.R. (2018). Cell walls of the dimorphic fungal pathogens Sporothrix schenckii and Sporothrix brasiliensis exhibit bilaminate structures and sloughing of extensive and intact layers. PLoS Negl. Trop. Dis..

[b0040] Moebius N., Üzüm Z., Dijksterhuis J., Lackner G., Hertweck C. (2014). Active invasion of bacteria into living fungal cells. Elife.

[b0045] Prados-Rosales R., Baena A., Martinez L.R., Luque-Garcia J., Kalscheuer R., Veeraraghavan U., Camara C., Nosanchuk J.D., Besra G.S., Chen B. (2011). Mycobacteria release active membrane vesicles that modulate immune responses in a TLR2-dependent manner in mice. J. Clin. Invest..

[b0050] Rivera J., Cordero R.J., Nakouzi A.S., Frases S., Nicola A., Casadevall A. (2020). Bacillus anthracis produces membrane-derived vesicles containing biologically active toxins. Proc. Natl. Acad. Sci. U. S. A..

[b0055] Rodrigues M.L., Nimrichter L., Oliveira D.L., Frases S., Miranda K., Zaragoza O., Alvarez M., Nakouzi A., Feldmesser M., Casadevall A. (2007). Vesicular polysaccharide export in Cryptococcus neoformans is a eukaryotic solution to the problem of fungal trans-cell wall transport. Eukaryot. Cell.

[b0060] Verstrepen K.J., Reynolds T.B., Fink G.R. (2004). Origins of variation in the fungal cell surface. Nat. Rev. Microbiol..

[b0065] Walker L.A., Sood P., Lenardon M.D., Milne G., Olson J., Jensen G., Wolf J., Casadevall A., Adler-Moore J., Gow N.A.R. (2018). The viscoelastic properties of the fungal cell wall allow traffic of AmBisome as intact liposome vesicles. MBio.

[b0070] Zhao L., Schaefer D., Xu H., Modi S.J., LaCourse W.R., Marten M.R. (2005). Elastic properties of the cell wall of Aspergillus nidulans studied with atomic force microscopy. Biotechnol. Prog..

